# Genetic diversity of the NE Atlantic sea urchin *Strongylocentrotus droebachiensis* unveils chaotic genetic patchiness possibly linked to local selective pressure

**DOI:** 10.1007/s00227-015-2801-y

**Published:** 2016-01-22

**Authors:** K. M. Norderhaug, M. B. Anglès d’Auriac, C. W. Fagerli, H. Gundersen, H. Christie, K. Dahl, A. Hobæk

**Affiliations:** Norwegian Institute for Water Research (NIVA), Gaustadallèen 21, 0349 Oslo, Norway; Department of Biosciences, University of Oslo, Blindern, P.O. Box 1066, 0316 Oslo, Norway; Department of Bioscience, Marine Diversity and Experimental Ecology, University of Aarhus, Frederiksborgvej 399, 4000 Roskilde, Denmark; Norwegian Institute for Water Research (NIVA), Region West, Thormøhlensgt. 53D, 5006 Bergen, Norway; Department of Biology, University of Bergen, P.O. Box 7803, 5020 Bergen, Norway

## Abstract

We compared the genetic differentiation in the green sea urchin *Strongylocentrotus droebachiensis* from discrete populations on the NE Atlantic coast. By using eight recently developed microsatellite markers, genetic structure was compared between populations from the Danish Strait in the south to the Barents Sea in the north (56–79°N). Urchins are spread by pelagic larvae and may be transported long distances by northwards-going ocean currents. Two main superimposed patterns were identified. The first showed a subtle but significant genetic differentiation from the southernmost to the northernmost of the studied populations and could be explained by an isolation by distance model. The second pattern included two coastal populations in mid-Norway (65°N), NH and NS, as well as the northernmost population of continental Norway (71°N) FV. They showed a high degree of differentiation from all other populations. The explanation to the second pattern is most likely chaotic genetic patchiness caused by introgression from another species, *S. pallidus,* into *S. droebachiensis* resulting from selective pressure. Ongoing sea urchin collapse and kelp forests recovery are observed in the area of NH, NS and FV populations. High gene flow between populations spanning more than 22° in latitude suggests a high risk of new grazing events to occur rapidly in the future if conditions for sea urchins are favourable. On the other hand, the possibility of hybridization in association with collapsing populations may be used as an early warning indicator for monitoring purposes.

**Electronic supplementary material:**

The online version of this article (doi:10.1007/s00227-015-2801-y) contains supplementary material, which is available to authorized users.

## Introduction

The green sea urchin *Strongylocentrotus droebachiensis* (O.F. Müller 1776) (Echinoidea) is a key species with severe impact on coastal ecosystems. By large-scale overgrazing of kelp forests in the Pacific (Estes et al. 1998), NW Atlantic (Steneck et al. 2004) and NE Atlantic (Norderhaug and Christie 2009), it has profound ecological and economic importance. Kelp forests are highly productive (Pedersen et al. 2012) and diverse (Norderhaug et al. 2012) systems and deliver ecosystem services including habitats (Norderhaug et al. 2002), feeding grounds (Norderhaug et al. 2005) and nursery areas (Godø et al. 1989), while sea urchin-dominated barren grounds are low-productive marine deserts (Ling et al. 2015).

*S. droebachiensis* has a broad Arctic-boreal distribution in the Atlantic and Pacific (Scheibling and Hatcher 2013). In the NE Atlantic, it is distributed from Denmark in the south [56°N, (Dahl et al. 2005)] to Svalbard (79°N, Gulliksen and Sandnes 1980) and Novaja Semlja (Propp 1977) in the north. A large cohesive barren ground area (up to 2000 km^2^) has dominated the coast of mid-Norway and North Norway and Russian Kola coast for more than four decades as a result of a sudden growth in the sea urchin populations (Sivertsen 1997). Observations from fishermen from around 1970 suggest that this barren was created quickly when urchins in high densities formed fronts and grazed all kelp forests between 63° 30′N on the Norway coast and beyond 71°N into Russian water (Norderhaug and Christie 2009). Small discrete urchin populations have also dominated fjords as far south as the Gullmarsfjord at the west coast of Sweden (back to the nineteenth century (Norderhaug and Christie 2009). Also, in the Danish Strait (at approximately 56°N), boulder reefs deeper than 13–15 m are heavily grazed by *S. droebachiensis* (Dahl et al. 2005). These *S. droebachiensis* populations thus prevail more than nine degrees south of the population on the Norwegian coast.

**Fig. 1:**
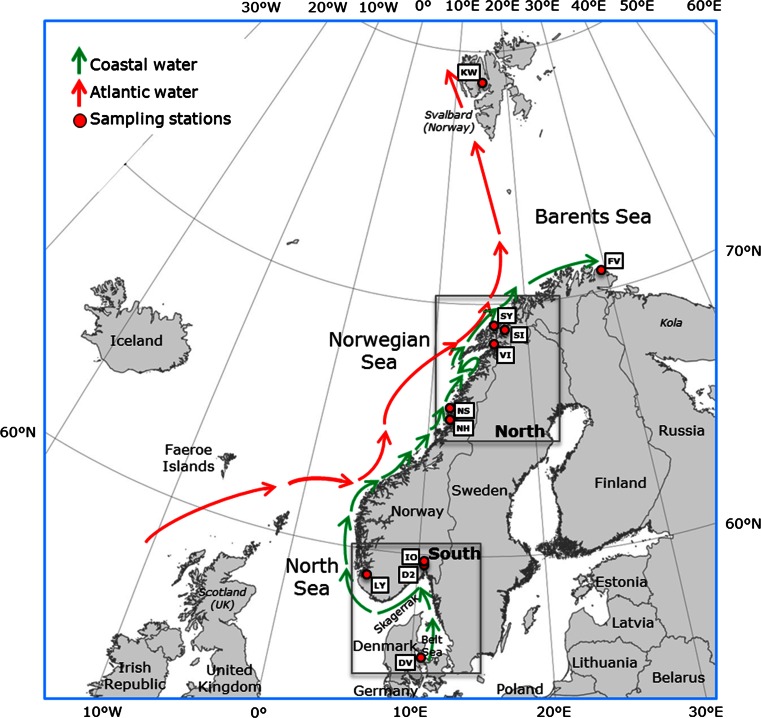
Map of the study area including sampling stations and a simplified illustration of the dominating currents along the coast from the Danish Belt Sea to the Barents Sea. *Green arrows* indicate the northbound coastal current, and *red arrows* indicate ocean currents from the NE Atlantic. *Red circles* and *white boxes* show the position and codes of the sample stations. See Table 1 for explanation of the codes. Oslofjorden (IO and D2), Lysefjorden (LY), Salangen (SI and SY) and Isfjorden (KW) are sill fjords, whereas the fjords represented by NH, NS VI and FV are open fjords/coastal areas. DV is located in the Danish Straits. The *squares* indicates groups (South and North) used in Migrate analysis

The distribution of benthic species with pelagic propagules is typically driven by the physical environment and how it has changed historically (Hoarau et al. 2007). Circulation patterns may be important for gene flow between urchin populations, carrying larvae from source to sink populations. The main current direction is from the south North Sea and Denmark and changes direction into the west flowing coastal current along the Norwegian Skagerrak coast which follows the coast northwards before it divides into two main currents heading north to Svalbard and east along the Barents Sea coast (Fig. 1; Sætre 2007). Tidal currents moving water in and out from the fjord every day (Sætre and Aure 2007) have the potential of carrying larvae from fjord to coastal water. Ocean currents may provide transport corridors between discrete populations in open marine water. However, large distances may represent barriers to dispersal of pelagic larvae between populations and result in high degree of isolation between populations of benthic marine animals (Reisser et al. 2011). Gyres along the Norwegian coast increase the retention time and may isolate populations locally. Also, echinoderms are sensitive to low salinity; hence, outflowing brackish surface water may represent barriers against dispersal of larvae out of fjords (Scheibling and Hatcher 2013). It has been hypothesized that urchin populations in Norwegian sill fjords may have been trapped in cold, saline deep water inside fjord basins and isolated since the last ice age (Fredriksen 1999). Historical geographical barriers created during the ice ages are important for the current distribution of shallow water marine species with pelagic dispersal propagules (Hu et al. 2011; Reisser et al. 2011).

To what extent these small, southernmost populations supply larvae to populations further north is unknown. *S. droebachiensis* has high fecundity and is a free spawner with a planktonic larval stage lasting 4–21 weeks (Strathmann 1978; Hart and Scheibling 1988; Metaxas 2013); the dispersal potential is large exceeding 1000 km on the coasts of Nova Scotia despite relatively slow surface currents (Addison and Hart 2004). As the average planktonic larval stage duration for the species along the Norwegian coast is reported to be 16 weeks (Fagerli et al. 2013), high dispersal potential is also expected for NE Atlantic *S. droebachiensis*. Mature females are usually found February to April, spawning peaks in March, and the main settling period is during summer (Fagerli et al. 2013). In a study of population genetic structure of *S. droebachiensis* by the use of microsatellites, Addison and Hart (2004) found generally little differentiation between populations along the NW Atlantic coast, but considerable differentiation between one analysed population from the NE (North Norway) and populations in the NW Atlantic. They found no evidence of gene flow across the Atlantic, and the genetic difference between the NE and the NW Atlantic was even larger than the difference between populations in the NW Atlantic and populations from the Pacific coast. Marks et al. (2008) found indication of first stage speciation between *S. droebachiensis* from the NW and NE Atlantic. The NW Atlantic populations seem more closely related to populations from the N Pacific than NE Atlantic, but gene flow studies from the NE Atlantic are lacking.

The main aims of this study were to analyse genetic diversity and gene flow among *S. droebachiensis* populations in the NE Atlantic and assess possible links to physical features like ocean currents and fjord topography, as well as observed changes in this species distribution. Microsatellites recently developed for studying NE Atlantic populations were used in this study (Anglès d’Auriac et al. 2014). We compared differentiation across the species’ geographical distribution in the NE Atlantic (i.e. from Denmark in the south to Svalbard in the north) and from sill fjords, open fjords and coastal populations. Increased knowledge of gene flow between urchin populations and detection of genetic patterns disruption is important to assess risk for future grazing events and is thus highly relevant for the management of NE Atlantic coastal areas. For instance, a strong gene flow may show rapid recruitment ability and grazing events in the future. By contrast, detection of localized disruption of such a gene flow might be informative pertaining to ongoing ecosystem changes possibly affecting the equilibrium of the species and therefore associated grazing events along the Norwegian coast.

## Materials and methods

### Area of investigation and field sampling

The study area was chosen to cover the NE Atlantic distribution range of *Strongylocentrotus droebachiensis*. We included stations from the open coast as well as inside sill fjords and open fjords. Samples were primarily taken from 2 to 22 m depth, as abundant urchins were only found deep at the southern populations (Table 1). Sea urchins were collected by SCUBA divers at each of the 11 stations: 7 from open coastal areas or fjord mouths (DV, D2, NH, NS, VI, SY, FV, Table 1) and 4 from inside sills in fjords (IO, LY, SI, KW). Gonad material from 30 to 40 large urchins was sampled and fixed on site by ethanol. Sea urchins from Lysefjorden were collected and put in a styrofoam box and transported express to the laboratory in Oslo where samples were preserved within 8 h after they were collected.

**Table 1:** Stations used in the study. Station name, station code, sampling depth (metre), urchin size (average diameter ± standard deviation), position (WGS1984, latitude and longitude), distance (km) downstream the Vejrø station and number of analysed individuals per population (N) are shown

Sea	Area	Station	Code	Depth	Size	Lat. (°N)	Long. (°E)	Distance	*N*
Belt Sea	Denmark	Vejrø	DV	20	40.1 (5.5)	55.93862	10.76797	0	30
Skagerrak	Oslo fjord	Drøbak	D2	20	22.1 (1.9)	59.66278	10.62596	430	28
Skagerrak	Oslo fjord	Svestad	IO	20	30.0 (6.3)	59.77624	10.59165	440	30
North Sea	Rogaland	Lysefjorden	LY	22	48.0 (7.6)	59.00875	6.315985	815	30
Norwegian Sea	Torghatten	Helløya	NH	5	46.1 (5.6)	65.38705	12.0149	1645	30
Norwegian Sea	Vega, nord	Skogsholmen	NS	5	38.4 (5.4)	65.81499	12.04164	2035	25
Norwegian Sea	Vestfjord	Tysfjord	VI	2	38.3 (5.2)	68.23613	16.22802	2110	30
Norwegian Sea	Salangen	Løksefjorden	SI	5	35.3 (4.6)	68.91894	17.70712	2380	29
Norwegian Sea	Salangen	Meløyvær	SY	2	48.2 (5.1)	69.07234	16.47666	2335	29
Barents Sea	Kongsfjord	Veidnes	FV	5	52.1 (5.3)	70.72	29.44	2865	30
Barents Sea	Svalbard	Kapp Wijk	KW	5	25.9 (3.1)	78.6	15.1667	3335	30

### Geographical distance calculations

Geographical distances were calculated as the distance from the southernmost population located close to the Vejrø Island in Denmark to all other stations further north. Since, to our knowledge, GIS maps (shape files or other) of ocean currents of the NE Atlantic do not exist, we created shape files in a GIS made from approximate, manually drawn paths following the main currents across Kattegat, along the Swedish and Norwegian coast, and crossing the Barents Sea to Svalbard (Fig. 1) based on a published map from Sætre and Aure (2007). Thus, the geographical distances calculated are not exact and the actual travelling distance may well be much longer, taking into account smaller currents and gyres and wind-driven deviations that may prolong the actual travel distance considerably. The geographical distance can thus be regarded as the least possible travel distance from Denmark and northwards following the main coastal currents and is assumed to be of sufficient precision for the purpose of this study.

### Laboratory analysis

Rapid crude DNA extraction using 96 % ethanol-preserved gonad tissue was performed as described in Anglès d’Auriac et al. (2014). A total of 321 individuals were analysed using 10 microsatellites loci specifically developed for the Northeast Atlantic *S. droebachiensis* (Anglès d’Auriac et al. 2014). Among these microsatellites, Strdro-837 and Strdro-849 acted as polysomic and were not included in the analysis. Briefly, a 3-primer PCR approach was used using a M13 tail for the forward primers as well as labelled forward M13 primers in addition to the reverse primers. Simplex PCR amplifications were performed using iProof mastermix (Bio-Rad, Hercules, CA, USA) and a CFX96 thermocycler (Bio-Rad). The amplification products of up to four different microsatellite loci, each labelled with a different dye, were mixed for product size characterization using a 3730XL DNA analyser (Applied Biosystems, Foster City, CA, USA). Alleles were scored using GeneMapper software version 4.0 (Applied Biosystems).

Two populations, NH and NS, were found to be highly differentiated from the others. These were found in the middle of the sampling area. To exclude the possibility of this deviation resulting from sampling the wrong species, in particular *S. pallidus* (Gagnon and Gilkinson 1994), we sequenced part of the mitochondrial cytochrome oxydase I (COI) on three individuals from each NH and NS stations as well as from the Northernmost and Southernmost Norwegian stations, respectively, KW and D2. A 1056-bp COI fragment was amplified using the following primers: 5-ACACTTTATTTGATTTTTGG-3 (forward) and 5-CCCATTGAAAGAACGTAGTGAAAGTG-3 (reverse) described by Lee (2003); Balakirev et al. (2008). The phylogenetic analysis included 12 additional sequences from GenBank. PCR amplifications were performed using a CFX96 Bio-Rad thermocycler (Bio-Rad, Hercules, CA, USA) in 15 μl reaction volume containing 7.5 μl SsoFast or iProof mastermix (Bio-Rad), 0.1 μM of each primers and 2.5 μl sample (8 ng/μl DNA). Reaction volume was completed with sterile deionized water. PCR amplifications were carried out under the following conditions: a denaturing step for 2 min (SsoFast) or 30 s (iProof) at 98 °C, followed by 40 cycles of 98 °C for 10 s, 50 °C for 30 s and 72 °C for 40 s followed with a final extension at 72 °C for 2 min. Cycle sequencing was performed in both directions using amplification primers and BigDye Terminator version 3.1 kit (Life technologies, Applied Biosystems). One microlitre PCR template was used with 0.5 µl Terminator mix, 0.32 µl 10 µM forward or reverse primer, 1.75 µl Terminator 5X buffer in a final volume of 10 µl. Cycle sequencing was performed using an ABI 7500 qPCR machine (Life technologies, Applied Biosystems) as following: 96 °C for 1 min followed by 28 cycles of 96 °C for 10 s, 50 °C for 5 s and 60 °C for 4 min. Sequence purification was performed using BigDye XTerminator Purification kit (Life technologies, Applied Biosystems) adding to each PCR sample well 10 µl XTermination solution and 45 µl Sam solution, final volume of 65 µl. The PCR plate was then sealed and vortexed for 30 min prior to being processed by an ABI3730XL DNA analyser (Life technologies, Applied Biosystems). Trace files analyses and sequence alignments were performed using CodonCode Aligner version 5.1.5 (CodonCode Corporation), and the evolutionary history was inferred using the UPGMA method (unweighted pair group means analysis). The evolutionary distances were computed using the Kimura 2-parameter method [3] and are in the units of the number of base substitutions per site. The analysis involved 24 nucleotide sequences. All positions containing gaps and missing data were eliminated. There were a total of 771 positions in the final dataset. The analysis was conducted using MEGA version 6.06 (Tamura et al. 2013).

### Statistical analysis

Data files from GeneMapper were imported into Microsoft Excel (version 14) and formatted for analysis in GenAlEx (version 6.5.01 (Peakall and Smouse 2012). GenAlEx was used to calculate allele frequencies as well as for analysis of molecular variance (AMOVA; Excoffier et al. 1992) and principal coordinate analysis. This add-in was also used to export data for other programs, like MicroChecker [version 2.2.3 (Van Oosterhout et al. 2004)] which was applied for estimating null allele frequencies. We used Arlequin [version 3.5.1.2 (Excoffier and Lischer 2010)] to estimate deviations from Hardy–Weinberg equilibrium and linkage disequilibrium evaluation.

A search for substructures in genetic variation was conducted in Structure version 2.3.3 (Pritchard et al. 2000) implemented at https://lifeportal.uio.no/. The analysis was run with 10^6^ burn-in iterations followed by 10^6^ Markov chain Monte Carlo steps, applying an admixture model with correlated allele frequencies utilizing location information (LOCPRIOR and LOCISPOP set to 1, but USEPOPINFO set to 0). Results of 10 independent runs with *K* = 1–10 clusters were input to Structure Harvester (Earl and vonHoldt 2012) to evaluate probabilities of *K* with the Evanno et al. (2005) method and then summarized in CLUMPP version 1.1.2 (Jakobsson and Rosenberg 2007) and finally plotted with Distruct (Rosenberg 2004).

Most loci showed considerable heterozygote deficiencies, which resulted in significant departures from HW expectations. This involved all loci except Strdro-97 and 7209 and indicated the possible presence of null alleles although using simplex PCR reduces the possible occurrence of false-negative loci amplification results. We applied corrections as suggested by the Brookfield1 algorithm (Brookfield 1996) implemented in MicroChecker (Van Oosterhout et al. 2004), which entails replacing one of two alleles in a homozygous state with a missing value. We chose the Brookfield1 approach as being the most conservative, i.e. requiring the least manipulation of data. This correction removed most of the HW deviations, although some remained. Later analyses were performed on the corrected as well as the original data. We observed linkage disequilibria between certain loci in some populations, but no pairs of loci showed signs of linkage across all populations. Thus, all loci were retained in further analyses.

Genetic differentiation between populations was estimated as Jost’s *D* (obtained in GenAlEx) as well as *F*_ST_, based on null-allele-corrected allele frequencies. For Jost’s *D*, we used null allele corrections as decribed above, while *F*_ST_ values were computed in the FreeNA software (Chapuis and Estoup 2007). This software estimates null allele frequencies using the EM algorithm (Dempster et al. 1977) and then calculates an *F*_ST_ matrix applying the ENA correction (Chapuis and Estoup 2007) based on adjusted frequencies of visible alleles (thus disregarding the null alleles).

Effects of null alleles on inbreeding (fixation index, *F*_IS_) were analysed with INEST version 2.0 (Chybicki and Burczyk 2009), applying the default Bayesian approach using 300,000 steps, sampling every 100 steps and discarding the first 30,000 steps as burn-in.

Testing for selection on the eight loci was conducted in BayeScan version 2.1 (Foll and Gaggiotti 2008), applying default settings. Data were corrected for null alleles prior to these analyses, and only Group 1 populations and individuals as identified by Structure (see ‘Results’ section) were included.

We estimated gene flow between geographical regions using Migrate version 3.36.11 (Beerli 2006, 2009). This approach is based on a coalescence model and provides mutation-scaled estimates of effective population sizes (*N*_e_) and migration parameters between populations under specified dispersal scenarios (models) using genetic data. Posterior distributions of parameters (effective population sizes and migration rates) were generated by Bayesian inference using Markov Chain Monte Carlo runs. Basically, we ran three migration models: migration from A to B only, from B to A only or both ways. Posterior model probabilities were compared using Bayes factors. Parameters were free to vary over intervals specified as priors, set for each run. We applied uniform prior distributions of effective population size Θ from 0 to 200 and for migration rate M from 0 to 100. Mutation rate was constant over all loci. We applied a static heating scheme of 4 chains with temperatures proposed by the program (1, 1.5, 3 and 10^6^ degrees), with swapping intervals set at 10. Only Group 1 individuals (as identified by the Structure analysis) were included in the datasets, since Group 2 individuals only occurred in a few areas, whereas Group 1 was present at all but one sampling stations. Since there was evidence for selection on five out of eight loci (see ‘Results’ section from BayeScan runs), only three loci were included in the dataset (Strdro-97-R, Strdro-7209 and Strdro-5563). We pooled the DV, IO, D2 and LY samples into one ‘South’ group and individuals from NH, VI, SI and SY into one ‘North’ group (Fig. 1).

## Results

### Genetic diversity and differentiation

All loci were polymorphic, with between 11 (Strdro-97 and 7209) and 26 (Strdro-5563) alleles detected per locus (Table 2). Populations did not differ markedly in allelic richness, with average number of alleles ranging between 7.12 and 10.0 per locus (Table 3). The number of private alleles ranged from none at stations DV and IO to 6 at station KW (=average 0.75 per locus, Table 3). Genetic diversity (expected heterozygosity) varied only slightly, from the highest estimate in population NH (0.69) to the lowest in SI (0.56). Observed heterozygosity was highest in LY (0.43) and lowest in KW (0.3) (Table 3).

**Table 2:** Characterization of 8 microsatellite loci for *S. droebachiensis* using 321 individuals

Locus	Size range (bp)*	*A*	*N*	*H*_o_	*H*_e_
Strdro-97	159–189	11	320	0.27	0.32
Strdro-1051	186–220	17	295	0.35	0.68
Strdro-4147	75–115	19	282	0.29	0.76
Strdro-7209	128–162	11	281	0.25	0.27
Strdro-5563	165–269	26	287	0.30	0.65
Strdro-1356	164–254	25	306	0.56	0.88
Strdro-7412	92–124	15	308	0.71	0.82
Strdro-5950	96–152	22	274	0.39	0.86

Number of alleles (*A*), number of individuals amplified (*N*), observed heterozygosity (*H*_o_), expected heterozygosity (*H*_e_), * including the 18-bp M13 forward tail

**Table 3:** Allelic richness, private alleles, expected and observed heterozygosity (genetic diversity) within populations

Population	DV	D2	IO	LY	NH	NS	VI	SI	SY	FV	KW
Total no. of Alleles	60	61	66	60	80	69	71	57	73	74	65
Mean no. Alleles	7.500	7.625	8.250	7.500	10.000	8.625	8.875	7.125	9.125	9.250	8.125
	(1.336)	(1.499)	(1.656)	(1.086)	(0.886)	(1.238)	(1.368)	(1.125)	(1.552)	(1.191)	(1.093)
Total no. Private Alleles	0	1	0	4	2	3	5	3	2	3	6
Mean no. Private Alleles	0.000	0.125	0.000	0.500	0.250	0.375	0.625	0.375	0.250	0.375	0.750
	(0.000)	(0.125)	(0.000)	(0.189)	(0.164)	(0.263)	(0.183)	(0.263)	(0.164)	(0.263)	(0.250)
Expected heterozygosity	0.601	0.593	0.607	0.597	0.665	0.644	0.605	0.524	0.615	0.648	0.562
	(0.085)	(0.102)	(0.101)	(0.088)	(0.071)	(0.075)	(0.075)	(0.080)	(0.094)	(0.078)	(0.105)
Observed heterozygosity	0.398	0.398	0.409	0.431	0.404	0.425	0.395	0.308	0.385	0.396	0.299
	(0.090)	(0.075)	(0.062)	(0.104)	(0.046)	(0.082)	(0.078)	(0.065)	(0.064)	(0.052)	(0.068)

Standard errors in parentheses

Genetic differentiation between populations is here presented as estimates of *F*_ST_ and Jost’s *D*_EST_ estimates (Table 3). Although several populations differed significantly from each other, the genetic differentiation between populations was generally low (*D*_EST_ < 0.06) over a wide geographical range (56–79°N). However, two coastal stations (NH Helløya and NS Skogsholmen at 65°N in the middle of the distribution area) stood apart, showing a much higher level of divergence (*D*_EST_ 0.11–0.32, Table 4). Estimates of *F*_ST_ were generally lower than *D*_EST_, ranging from 0.01 to 0.10. Nonetheless, *F*_ST_ estimates also differed significantly from zero in exactly the same pairwise comparisons as *D*_EST_.

**Table 4:** Genetic differentiation between populations and sub-populations

	D2	DV	FV2	FV1	KW	NH2	NS	SI	SY	IO	LY	VI
D2		−0.007	**0.1053**	0.0217	0.0057	**0.1192**	**0.1316**	**0.0295**	**0.0239**	0.0018	0.0031	0.0075
DV	−0.006		**0.0948**	**0.0381**	**0.0173**	**0.1081**	**0.1200**	**0.0336**	**0.0265**	0.0020	−0.0013	**0.0111**
FV2	**0.251**	**0.217**		**0.1476**	**0.1090**	−0.0033	0.0038	**0.0959**	**0.0667**	**0.1103**	**0.1103**	**0.0771**
FV1	**0.032**	0.038	**0.293**		0.0125	**0.1754**	**0.1891**	0.0130	0.0410	**0.0343**	**0.0409**	0.0247
KW	−0.004	0.009	**0.230**	0.004		**0.1304**	**0.1378**	0.0071	0.0069	**0.0113**	**0.0189**	0.0054
NH2	**0.288**	**0.253**	−0.019	**0.351**	**0.288**		−0.0028	**0.1329**	**0.0941**	**0.1191**	**0.1289**	**0.1051**
NS	**0.321**	**0.279**	−0.004	**0.375**	**0.310**	−0.023		**0.1377**	**0.0967**	**0.1293**	**0.1401**	**0.1154**
SI	**0.037**	**0.045**	0.222	0.012	0.003	**0.298**	**0.309**		0.0044	**0.0377**	**0.0371**	0.0039
SY	**0.030**	**0.039**	**0.168**	**0.065**	0.009	**0.230**	**0.250**	0.008		**0.0233**	**0.0328**	0.0064
IO	−0.002	0.001	**0.237**	**0.061**	0.014	**0.271**	**0.308**	**0.059**	**0.035**		−0.0005	**0.0249**
LY	0.007	−0.005	**0.229**	**0.061**	**0.027**	**0.281**	**0.317**	**0.049**	**0.056**	0.001		**0.0184**
VI	0.008	0.012	**0.189**	0.036	0.000	**0.252**	**0.278**	0.003	0.002	**0.034**	0.021	

*F*_ST_ estimates are given above the diagonal, and Jost’s *D*_EST_ below. Estimates significantly different from zero are set in bold. *F*_ST_ with 95 % confidence limits were estimated in FreeNA with the ENA method for null allele correction. *D*_EST_ values were calculated in GenAlEx on null-allele-corrected data, with significance values estimated by 999 random permutations. The significance level for *D*_EST_ was adjusted to 0.017 using the false discovery rate (10 comparisons for each station). Sub-population NH1 is not included (only 5 individuals)

**Fig. 2:**
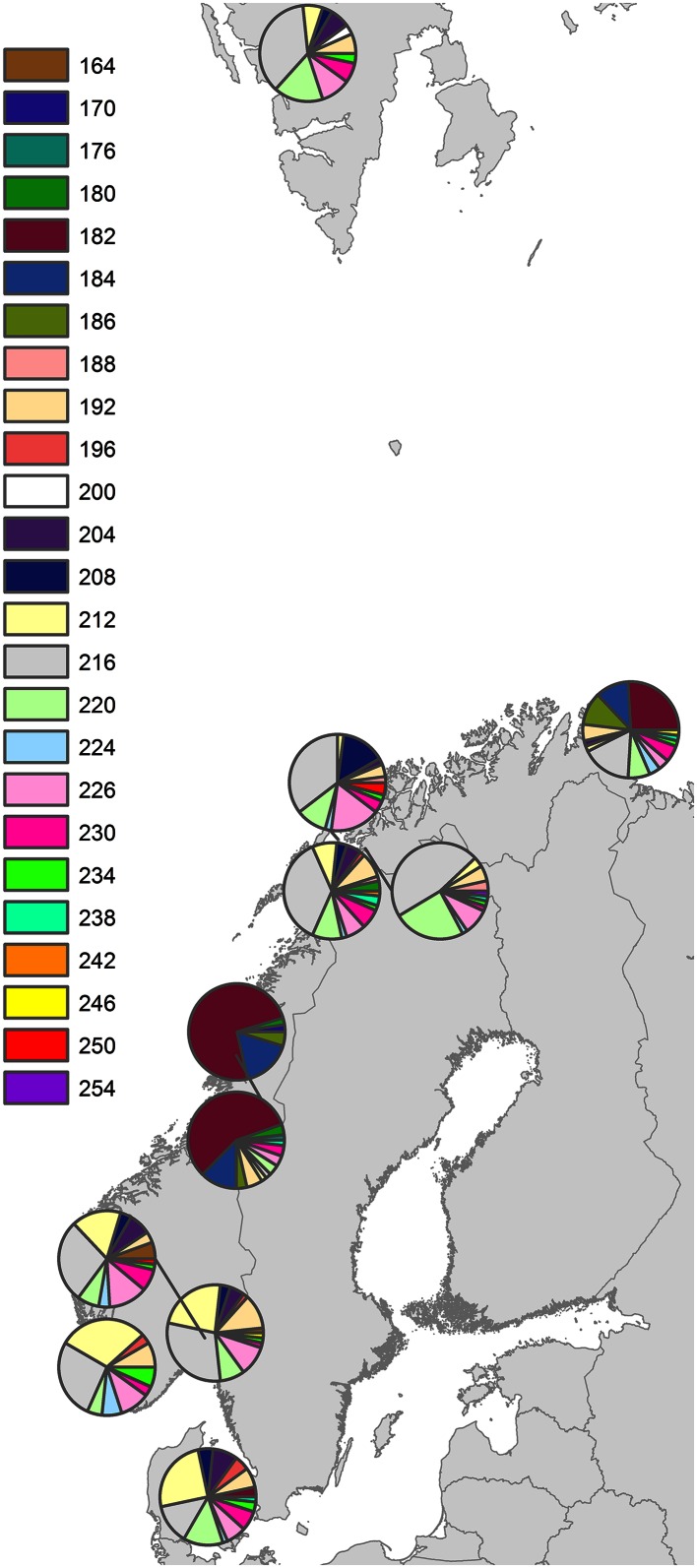
Allele frequencies by populations shown for locus Strdro-1356. See Supplement 1 for the other 7 loci

Only five loci (Strdro-97, 1356, 4147, 5563 and 7209) differentiated significantly (*p* < 0.05) between populations in an overall calculation, resulting in an overall *D*_EST_ of 0.085 (SE 0.050). Among these five loci, Strdro-97 and 7209 contributed little to differentiation (*D*_EST_ 0.009 and 0.012), while the remaining three loci showed stronger differentiation (*D*_EST_ 0.116–0.471). This was due to marked differences in allele frequencies at these loci, particularly at Strdro-5563 and 1356. Figure 2 shows allele frequencies on the latter locus, while similar figures for other loci are given in Supplementary Information.

*F*_IS_ estimates in INEST became lower in all populations when null alleles were allowed in the calculations, and this model was preferred over the alternative by the DIC criterion in all cases. Estimates of *F*_IS_ varied from 0.022 (population LY) to 0.22 (population IO, Table 5). However, the posterior 95 % probability intervals included zero for all population estimates. Thus, all *F*_IS_ estimates were not significantly different from zero, indicating no significant inbreeding in these populations. Increasing the number of MCMC steps from 300,000 to 600,000 had negligible effects on the estimates of *F*_IS_ as well as the posterior distributions.

**Table 5:** Estimates of the fixation index (*F*_IS_) in INEST 2.0

Population	Number of individuals	*F*_IS_ w/o null alleles	*F*_IS_ with null alleles	DIC without null alleles	DIC with null alleles	95 % posterior probability interval
DV	30	0.3454	0.0597	1195.5	1172.5	0–0.3027
D2	28	0.3380	0.1346	1179.8	1177.8	0–0.3027
IO	30	0.3499	0.2198	1262.8	1259.7	0–0.3615
LY	30	0.2781	0.0220	1213.3	1170.7	0–0.2250
NH2	25	0.4027	0.1115	1125.0	1112.8	0–0.3007
NS	25	0.3725	0.1141	1120.5	1105.8	0–0.2830
VI	30	0.3499	0.0848	1289.4	1267.8	0–0.2046
SI	29	0.4129	0.0817	1049.3	1041.9	0–0.2552
SY	29	0.3910	0.1936	1268.9	1262.5	0–0.3645
FV1	14	0.2501	0.1068	489.3	484.0	0–0.2759
FV2	16	0.3635	0.1367	704.4	701.8	0–0.3323
KW	30	0.4821	0.1192	1169.9	1151.9	0–0.3450

Estimates are given for models with and without considering null alleles. The deviance information criterion (DIC) for both models is also given. The final column provides the 95 % posterior probability intervals for the model including null alleles

An analysis of molecular variance (AMOVA) with Group 1 animals partitioned most of the genetic variance (96.4 %) to within populations and the remaining 3.6 % among populations (Table 6). Variation within individuals was suppressed in this analysis. AMOVA results did not change with null-allele-corrected data.

**Table 6:** Analysis of molecular variance (AMOVA) populations within Group 1

Source	*df*	SS	MS	Est. Var.	%
Among populations	10	74.951	7.495	0.099	3.6
Within populations	531	1424.004	2.682	2.6882	96.4
Total	541	1498.956		2.781	100.0

The rightmost column shows partitioning of genetic variance. Overall *F*_ST_ = 0.036 (*p* = 0.001)

**Fig. 3:**
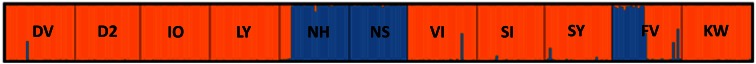
Clustering of 8-locus genotypes with *Structure* in two groups (*K* = 2 preferred by both log probability of *K* and by the Evanno method). Group 1, coloured in *orange*, gathers most individuals and populations, whereas group 2, coloured in *blue*, is found in NS, NH and about half of FV individuals

**Fig. 4:**
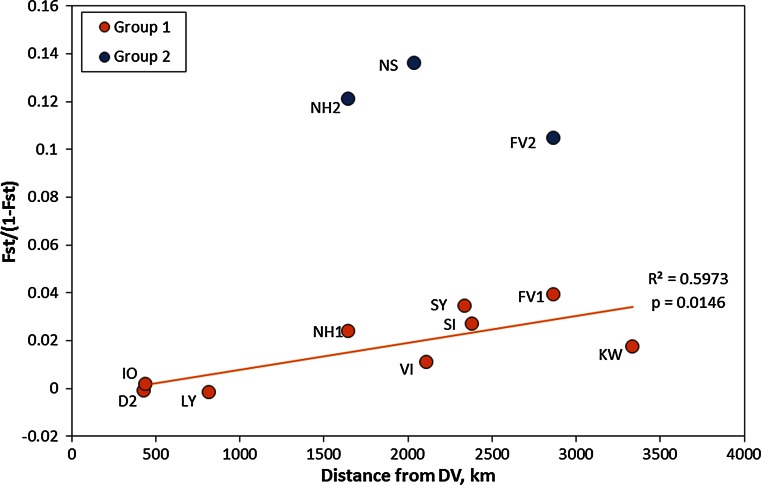
Genetic versus geographical distance. Scaled differentiation (*F*_ST_/(1 − *F*_ST_)) for each population compared with the southernmost Belt Sea (DV) population. Based on the Structure results shown in Fig. 3, NH and FV are both split in two, NH1, NH2, FV1 and FV2. Statistics are based on the linear regression between the two variables, with the intercept forced to 0 (Danish population, DV)

Analysis of population structure clearly indicated two subgroups (Fig. 3). With *K* = 2, the highest rate of change (delta *K* = 479.3; 32 times higher than any other step) and mean log probability of *K* levelled off at higher *K*. Concordant with the genetic differentiation estimates, one cluster dominated stations from south to north, except the NH and NS station in mid-Norway, which were mainly allocated to the second cluster, and the FV station where individuals were evenly divided among clusters (Fig. 4).

### Isolation by distance (IBD)

Based on the expectation that larval transport occurs primarily from south to north following the Norwegian coastal current, we plotted an estimate of genetic differentiation between the southernmost population from the Belt Sea (DV) and all other populations versus estimated distance between stations (Fig. 4). Linearized *F*_ST_ values (i.e. *F*_ST_/(1 − *F*_ST_)) were then plotted against estimated distances from the southernmost site DV, to test a hypothesis of IBD given an assumed dominant northwards transport of pelagic larvae.

### Species identity

**Fig. 5:**
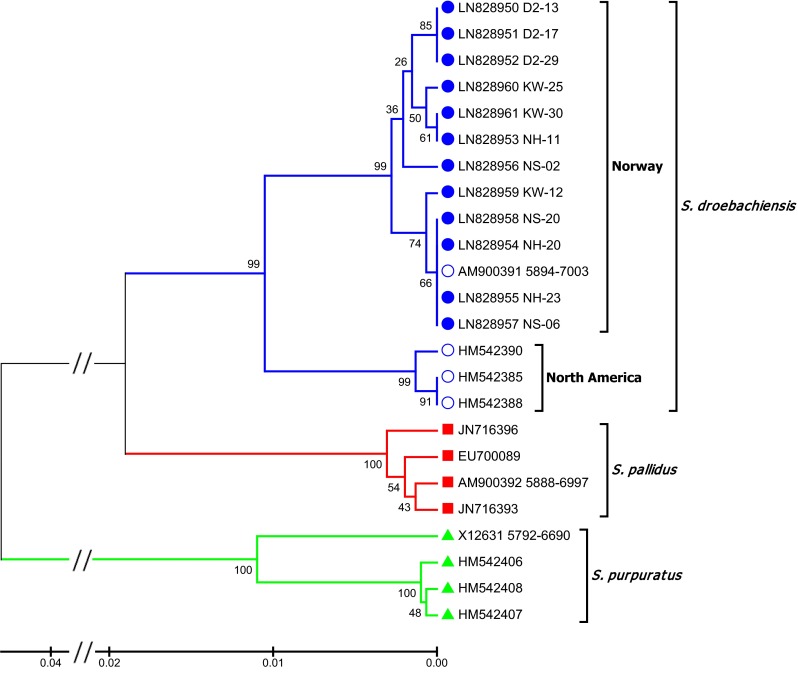
UPGMA (unweighted pair group means analysis) tree for the COI sequences using Kimura 2-parameter substitution model. The percentage of replicate trees in which the associated taxa clustered together in the bootstrap test (10,000 replicates) is shown next to the branches. The tree is drawn to scale, with branch lengths in the same units as those of the evolutionary distances used to infer the phylogenetic tree. In addition to the 12 *S. strongylocentrotus* individuals sequenced for this study shown by full blue circles, 12 sequences were obtained from GenBank: 4 *S. strongylocentrotus*, 4 *S. pallidus* and 4 *S. purpuratus* sequences. All access numbers are indicated in the figure

The *S. droebachiensis* mitochondrial partial COI sequences from this study are available at the European Nucleotide Archive: http://www.ebi.ac.uk/ena/data/view/LN828950-LN828961. Our sequences clustered with another sequence from Norway (positions 5894-7003 in GenBank accession number AM900391, Fig. 5). Three sequences from the NW Atlantic grouped in a separate clade within *S. droebachiensis*, while *S. pallidus* sequences formed a distinct sister clade to *S. droebachiensis*. Thus, there is no doubt about the maternal species affiliation of our study populations, whereas possible *S. pallidus* paternal hybridization cannot be concluded upon with this analysis alone.

### Gene flow in Migrate

Our main purpose in estimating gene flow was to test the hypothesis that *S. droebachiensis* larvae primarily disperse from south to north following the coastal current. Hence, the primary objective was to compare migration models, rather than obtain absolute estimates of number of migrants. For the composite North and South populations, we found that the model of northwards migration had a much higher probability than the alternatives (Table 7). We were unable to obtain satisfactory estimates of effective population size (Θ) the ‘North’ group, which showed considerable variation of the estimate across the prior space as reflected in the differences between modes, median and mean estimates (Table 8). By contrast, the estimates for the ‘South’ group and the migration rate converged well, with the corresponding estimates closely tied.

**Table 7:** Comparison of migration models between the composite populations ‘North’ and ‘South’ (see text for details of groupings)

Migration model	Raw thermodynamic	Bezier approximation	Harmonic mean	BF (Bezier)
North ↔ South	−4140.47	−1280.5	−332.94	2.7501E−78
North → South	−3091.46	−1101.91	−303.67	1
South → North	−6237.74	−1591.44	−294.11	2.5109E−213

MCMC long chains were 40,000 recorded steps. Three log probability scores are calculated by Migrate (raw thermodynamic, Bezier approximation, and Harmonic mean), while the final column gives the Bayes factors based on the Bezier approximation scores

**Table 8:** Estimates of mutation-scaled effective population sizes (Θ) and migration rates (M) for the preferred migration model (South → North) in Migrate, with an MCMC long chain length of 100,000 recorded steps

Estimate	Θ North	Θ South	M → nord
Mode	9.40	4.870	31.03
Median	55.93	5.000	31.50
Mean	71.20	4.820	30.20

The effective population size of the South’ group was clearly smaller than that of the ‘North’ group. Even so, the estimated mutation-scaled migration rate M from ‘South’ to ‘North’ was high (Table 8).

## Discussion

Two patterns superimposed could be distinguished of genetic differentiation of the NE Atlantic *S. droebachiensis* populations. The first pattern showed a consistent weak differentiation across its latitudinal distribution range which could be explained by an isolation by distance model (Fig. 4). This pattern is consistent with larvae being spread with ocean currents coming from the area of the southernmost population in the Danish Belt Sea area, going to the Norwegian Skagerrak coast and onwards by the coastal current turning west and north before dividing to the west coast of Svalbard and East Finnmark, respectively. The Danish (and southern Norwegian populations) may be fed with larvae from reef populations in the southeast North Sea and Danish Skagerrak. Parts of the current along the Danish Skagerrak coast turns south and penetrates into the Belt Sea area on deep water, while parts mix with Baltic water and later becomes the Norwegian coastal current.

This study thus supports the hypothesis that urchin populations in fjords and on the coast are generally not isolated, rather there seems to be relatively high gene flow from southern to northern populations and further into east Finnmark and west Svalbard, respectively. Northwards-flowing currents transport larvae from population to population, at least occasionally. With a larval phase lasting on average 16 weeks (Fagerli et al. 2013), larvae may be carried long distances from the source population before settling, providing an effective northwards-directional gene flow in the studied geographical area. The degree of isolation was relatively small across a large geographical area (over 3300 km from 56 to 79°N). Approximately 96 and 4 % of the observed genetic variation was found within and between the populations included in Group 1, respectively (according to AMOVA, Table 6). This is in line with what was found by Addison and Hart (2004) who found no differentiation among populations from Atlantic Canada consistent with high level of genetic flow or a low rate of genetic drift. There were thus few signs of isolation of populations even inside sill fjords, suggesting that sea urchins are in general not isolated by fjord circulation in sill fjords as earlier hypothesized by Fredriksen (1999), but there is likely considerable supply of larvae from upstream areas. The reason why sea urchin populations are only being found inside fjords in south Norway therefore seems not to be a lack of supply of larvae, but rather unfavourable conditions outside the fjords. This is discussed further below. LY Lysefjorden population showed some indications of higher isolation. While it did not deviate much in the isolation by distance model (Fig. 4), it had a relatively high numbers of private alleles (Table 4). The Lysefjorden is characterized by a shallow sill (13 m) and very little water exchange with the outside fjord water (Aure et al. 2001). Urchins are only found on rather deep water (from 22 m and below), and this population show signs of being trapped in deep water for long periods by the fjord circulation, and the results may show signs of higher isolation compared to the coastal and other fjord populations. This may indicate that physical fjord features may isolate some populations more than others.

The second main differentiation pattern, to our surprise, showed that two coastal populations in mid-Norway (65°N), NH and NS, as well as the northernmost population of continental Norway (70°N) FV, were very different genetically from the other populations and showed high degree of isolation from all other populations. This was indicated both by population differentiation estimates (*D*_EST_, *R*_ST_) and by the clear indication of two distinct clusters in Structure. Punctual presence of high diversity patches in the midst of genetically homogenous populations of a species covering large geographical areas is not uncommon, especially among marine invertebrates (Larson and Julian 1999). This phenomenon, chaotic genetic patchiness, has also been described for sea urchin populations which have experienced collapse caused by disease outbreaks (Addison and Hart 2004). The apparent paradox of such a pattern showing high genetic diversity among collapsing populations may be associated with introgression (Harper et al. 2007). The COI sequences we obtained showed unequivocally that the NS and NH individuals had a *S. droebachiensis* maternal origin, whereas hybridization with males from the closely related species *S. pallidus* could not be eliminated. Indeed, it is known that ova of *S. droebachiensis* can be fertilized by sperm of *S. pallidus* giving viable hybrids with pigmentation resembling *S. droebachiensis* in the adult stage (Strathmann 1981), and asymmetric introgression from *S. pallidus* into *S. droebachiensis* was further demonstrated (Addison and Pogson 2009). Hybridization between *S. droebachiensis* and *S. pallidus* was also suggested as early as 1952 in three specimens from the Trondheims fjord by (Vasseur 1952). Hence, the most likely explanation for this high diversity observed in areas where *S. droebachiensis* populations are collapsing is that a high selection pressure has paved the way for asymmetric introgression from *S. pallidus* into *S. droebachiensis*. These sea urchin populations in mid-Norway are currently collapsing and retreating northwards. Fagerli et al. (2013) found low urchin settlement around NS Skogsholmen compared to Hammerfest (near the North Cape at 71°N). The retreat is probably caused by ocean warming (Fagerli et al. 2013) and increasing predation on sea urchin recruits from northwards expanding *Cancer pagurus* and *Carcinus maenas* crabs as a result from warming (Fagerli et al. 2014). From the opposite side, invasive crabs from Russian waters, *Paralithodes camtschaticus,* invade the East Finnmark coast. This species was introduced to Kola (Russia) from the Pacific for marine cultivation purposes during the 1960s and has since the 1990s extended its range westwards into East Finnmark waters (Oug et al. 2011). Dense king crab populations in shallow water, collapse in sea urchin populations and recovery of kelp have been observed locally on the Russian coast during the last decade (Gudimov et al. 2003) and recently on the Norwegian coast (Christie and Gundersen 2014). While it is not known what have caused urchin collapse in this area, *S. droebachiensis* is a major prey for king crabs during spring when sea urchin recruits are settling (Pavlova 2009). If introgression is an early warning sign of urchin populations progressing towards collapse, larger areas shifting from sea urchin to kelp domination may be expected in the future.

The observed genetic differentiation in the NH and NS populations could also possibly be explained by hydrographical features: gyres south of Lofoten (i.e. the archipelago west of station VI in Fig. 1) may increase the retention time and trap pelagic larvae. South of Lofoten the current turns away from the coast and around Lofoten. South of Lofoten and inside the coastal current large gyres are formed (Aas 1994; Sætre 2007) that may isolate or delay larvae to such a degree that settlement only occurs locally. Isolation may be further increased because this is a very shallow area (locally referred to as ‘boot sea’ to illustrate that it is almost possible to take on boots and wade offshore). Larvae could also be brought to this part of the coast by ocean currents from Scotland, regularly or occasionally (Fig. 1). The Lofoten gyre or transport from Scottish water, however, cannot explain why the FV populations shared a similar differentiation and we find them thus less likely. Isolation would also be expected to lower diversity (as indicated for LY Lysefjorden) and not the observed increase in diversity.

The HWE disequilibrium we observe on the 321 samples with all 8 loci (Table 2) is very similar to that observed with the 96 samples used to establish the microsatellite method (Anglès d’Auriac et al. 2014), showing consistency in the observed disequilibrium. Such HWE disequilibrium has been previously observed in *S. droebachiensis* as for example with 3 of the 4 microsatellites used in a prior study (Addison and Hart 2004), hence suggesting that *S. droebachiensis* may naturally deviate from HWE as it has been reported to be the case for many other marine invertebrates (Brownlow et al. 2008).Our findings have important implications for the risk of future grazing events. Widely spread larvae from Danish Skagerrak and fjords in southern Norway imply a high risk of new grazing events which can be expected to occur rapidly in the future under favourable conditions for the green sea urchin. While climate variation cannot be managed, kelp forest resilience can. Therefore, emphasis should be focused on strengthening kelp forest state resilience to withstand future sea urchin blooms. Our findings can also be used for developing monitoring indicators. Regime shifts between sea urchin-dominated and kelp forest states which are being observed along the Norway coast occur typically suddenly and come as a surprise. Therefore, early warning signals for these types of events are difficult to identify (Möllmann et al. 2015). Our findings may provide an opportunity to develop tools for predicting and monitor sea urchin population collapse before and as they occur.

## Electronic supplementary material

Below is the link to the electronic supplementary material.

Supplementary material 1 (PDF 773 kb)
