# Supplementary material for: Genetic diversity of the NE Atlantic sea urchin Strongylocentrotus droebachiensis unveils chaotic genetic patchiness possibly linked to local selective pressure
Source: Mar Biol. 2016 Jan 22;163:36. doi: 10.1007/s00227-015-2801-y (PMC4722066; doi:10.1007/s00227-015-2801-y)
Supplement: Supplementary file 1 — Supplementary material 1 (PDF 773 kb) [file 227_2015_2801_MOESM1_ESM.pdf]

K. M. Norderhaug[1,2], M.B. Anglès d'Auriac[1], C. W. Fagerli[1], H. Gundersen[1], H. Christie[1], K. Dahl[3], A. Hobæk[4,5],

[1]Norwegian Institute for Water Research (NIVA), Gaustadallèen 21, 0349 Oslo, Norway.
[2]Department of Biosciences, University of Oslo, P.O. Box 1066, Blindern, 0316 Oslo, Norway.
[3]Department of Bioscience, Marine Diversity and Experimental Ecology, University of Aarhus, Frederiksborgvej 399, 4000 Roskilde, Denmark.
[4]Norwegian Institute for Water Research (NIVA), Region West, Thormøhlensgt. 53D, 5006 Bergen, Norway
[5]Department of Biology, University of Bergen, P.O. Box 7803, 5020 Bergen, Norway

Genetic diversity of the NE Atlantic sea urchin *Strongylocentrotus droebachiensis* unveils chaotic genetic patchiness possibly linked to local selective pressure

Supplement 1. Allele frequencies of the loci used in the study.

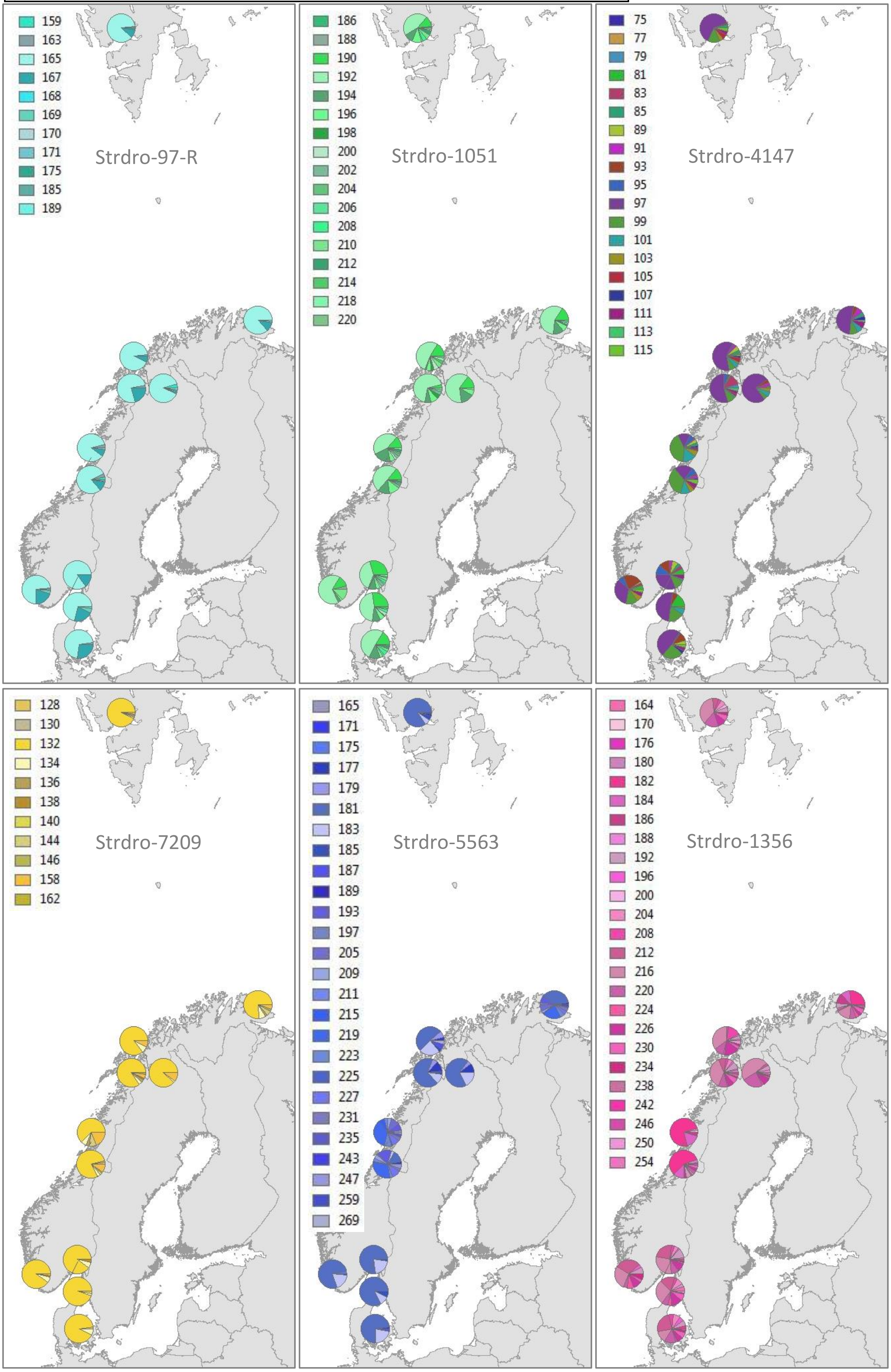

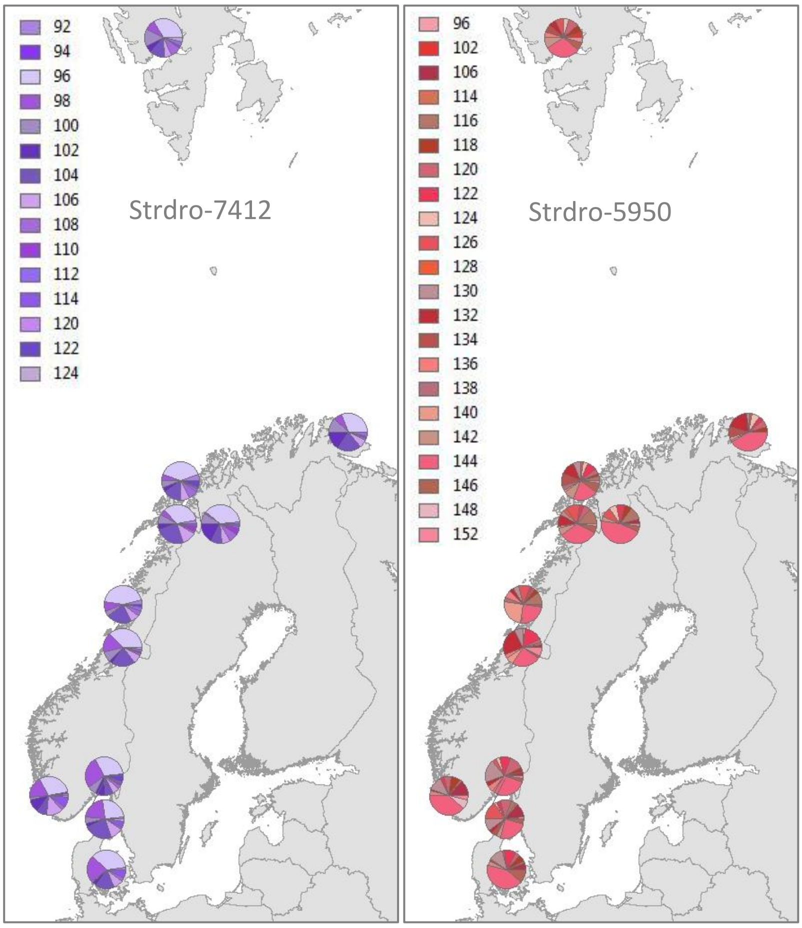
92
94
96
98
100
102
104
106
108
110
112
114
120
122
124
Strdro-7412
96
102
106
114
116
118
120
122
124
126
128
130
132
134
136
138
140
142
144
146
148
152
Strdro-5950
